# High‐Efficiency Broadband Achromatic Metadevice for Spin‐to‐Orbital Angular Momentum Conversion of Light in the Near‐Infrared

**DOI:** 10.1002/smsc.202300273

**Published:** 2024-02-13

**Authors:** Lingyun Xie, Hengyi Wan, Kai Ou, Junming Long, Zining Wang, Yuchao Wang, Hui Yang, Zeyong Wei, Zhanshan Wang, Xinbin Cheng

**Affiliations:** ^1^ Institute of Precision Optical Engineering School of Physics Science and Engineering Tongji University Shanghai 200092 China; ^2^ MOE Key Laboratory of Advanced Micro‐Structured Materials Shanghai 200092 China; ^3^ Shanghai Frontiers Science Center of Digital Optics Shanghai 200092 China; ^4^ College of Electronic and Information Engineering Tongji University Shanghai 200092 China; ^5^ School of Physics and Electronics Hunan Normal University Changsha 410081 China; ^6^ Shanghai Institute of Intelligent Science and Technology Tongji University Shanghai 200092 China

**Keywords:** achromatically focusing, control of dispersions, multifunctional metasurfaces, optical vortices, spin‐to‐orbit conversions

## Abstract

Spin‐orbital angular momentum conversion (SOC) of light has found applications in classical and quantum optics. However, the existing SOC elements suffer severe restrictions on broadband integrated applications at miniature scales, due to bulky configurations, single function, and failing to control the dispersion. Herein, a high‐efficiency broadband achromatic method for independently and elaborately engineering the dispersion and the SOC of light based on a cascaded metasurface device is proposed. The metadevice is capable of efficiently decoupling the SOC from the modulation of dispersion with high‐broadband focusing efficiency up to 75%. For the proof of concept, the generation of high‐efficiency achromatic‐focused and spin‐controlled optical vortices with switchable topological charge ($l^{\sigma =+1}=1$ and $l^{\sigma =-1}=2$) is successfully demonstrated. The presence of achromatically and highly concentrated optical vortices with tunable photonic angular momentum using spin as an optical knob makes the proposed ultracompact and multifunctional metadevice a promising platform for optical micromanipulation at nanoscale dimensions.

## Graphical abstract

Herein, a high‐efficiency broadband achromatic methodology for independently and elaborately engineering the dispersion and the spin‐orbital angular momentum conversion of light based on cascaded metasurfaces is proposed. The presence of achromatic optical vortices with tunable photonic angular momentum using spin as an optical knob makes the proposed ultracompact and multifunctional metadevice a promising platform for optical micromanipulation at nanoscale dimensions.

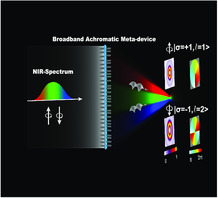

## 1 Introduction

Angular momentum (AM) of light includes spin angular momentum (SAM) and orbital angular momentum (OAM). It's well known that circularly polarized (CP) light carries SAM of ±ℏ per photon depending on its handedness (ℏ is reduced Planck's constant).^[^ ^1^, ^2^ ^]^ Optical vortex (OV) beams with helical wavefronts evolution in the form of exp(*ilφ*) around the azimuth, where *l* is the topological charge and can take any integer value, also carrying OAM of *l*ℏ per photon.^[^ ^3^, ^4^ ^]^ The twisted phase profile results in a doughnut‐shaped intensity profile. Such hollow beams carrying OAM have attracted much attention due to numerous applications in classical and quantum optics, including optical micromanipulation,^[^ ^5^, ^6^ ^]^ optical communication,^[^ ^7^, ^8^ ^]^ super‐resolution and edge‐enhanced detection,^[^ ^9^, ^10^ ^]^ and quantum entanglement.^[^ ^11^, ^12^ ^]^ Typically, in order to integrate the twisted helical mode into the wavefront of a paraxial beam for the generation of OV beams, spiral phase plates,^[^ ^13^ ^]^ spatial light modulators,^[^ ^14^ ^]^ pitch‐fork holograms,^[^ ^15^ ^]^ or laser mode conversion^[^ ^16^ ^]^ are used. Furthermore, to achieve circularly polarized OV beams carrying a total angular momentum (TAM) *L* = (*σ* + *l*) ℏ per photon (*σ* = ±1),^[^ ^17^ ^]^ the geometric phase‐based q‐plates are much more desired elements due to offering a direct connection between the SAM and OAM via SAM‐to‐OAM conversion (SOC). However, the output OAM is constrained to be a conjugate value of ±2*q*ℏ per photon.^[^ ^18^, ^19^ ^]^ The chromatic aberration, single function, and bulky configurations restrict their working bandwidth and use in emerging integrated optics operating at nanoscale dimensions.^[^ ^20^ ^]^

Optical metasurface, an artificially nanostructured pattern that strongly interacts with light, provides a fundamentally new methodology for efficient and accurate control of amplitude, phase, and polarization of light.^[^ ^21^, ^22^, ^23^, ^24^, ^25^ ^]^ Instead of shaping light waves through the propagation effect by conventional optics, it's capable of imparting a space‐variant wavefront change on an incident electromagnetic wave via an ultrathin planar nanostructure.^[^ ^26^, ^27^, ^28^, ^29^, ^30^, ^31^ ^]^ The metaoptics has revolutionized the concept of light control and brought unprecedented freedom over its conventional bulky and heavy counterpart in developing the ultracompact and integrated optical systems, such as miniature quarter‐wave‐plates,^[^ ^32^ ^]^ spin‐Hall devices^[^ ^33^, ^34^ ^]^ and quantum metasurfaces,^[^ ^35^, ^36^ ^]^ and nanoscale light source.^[^ ^37^, ^38^, ^39^ ^]^ The initial endeavors for miniature OV generators to achieve a helical beam with one specific topological charge for linearly and CP light are based on geometric phase‐based metasurface devices (metadevices).^[^ ^21^, ^40^ ^]^ Remarkable monochromatic metadevices for control of SOC has been proposed by engineering asymmetrical birefringent meta‐atoms.^[^ ^41^ ^]^ However, such metadevices generally yield a propagating OV instead of a focusing OV with concentrated photons at a predefined focal plane, which is highly desired for optical trapping and manipulation^[^ ^42^ ^]^ and optical detection.^[^ ^43^ ^]^ Therefore, their applications are seriously constrained when highly focused OV beams carrying different AMs are needed.^[^ ^44^, ^45^ ^]^

The focusing OV metadevice has attracted great attention via encoding a twisted helical wavefront profile onto a single metalens featuring the compact planar nature and diffraction‐limit focusing.^[^ ^46^, ^47^, ^48^, ^49^ ^]^ Compared with monochromatic or discrete multiwavelength achromatic ones,^[^ ^50^, ^51^, ^52^ ^]^ broadband achromatic metadevices (BAMs) are much more desired due to the more compact size and powerful functionalities in the visible and infrared spectrum.^[^ ^53^, ^54^, ^55^, ^56^, ^57^, ^58^ ^]^ Recently, polarization‐controlled BAMs have been well demonstrated,^[^ ^59^ ^]^ but fail to connect the SAM and OAM for on‐demand control of the SOC of light. Up to now, the broadband achromatic focusing for CP OV beams with switchable AM driven by the SOC has remained elusive. The major hurdle is the lack of a general methodology to efficiently decouple the SOC from the modulation of phase dispersion, to flexibly and independently engineer the different SOC states for multiplexing.

Recently, the emerging cascaded metasurfaces have expanded the freedom of light control, making compact, multifunctional, and high‐efficiency metadevices possible.^[^ ^60^, ^61^, ^62^ ^]^ In this work, we propose a general and high‐efficiency broadband achromatic method to independently engineer the dispersion and SOC of light for the first time. The design concept stems from a general approach in which cascaded metasurfaces can simultaneously implement achromatic focusing and spin‐multiplexing functionality. For the proof of concept, we successfully accomplish the BAM for control of the SOC through elaborately cascading a spin‐controlled metasurface and an irregular achromatic metasurface with focusing efficiency up to 90%. The efficient decoupling between the SOC and the control of dispersion ensures the realization of broadband achromatic‐focused OV beam with tunable topological charge number. The broadband efficiency of the BAM is up to 60%. The simulated results robustly confirm our approach. Since the proposed BAM is made of all‐dielectric silicon materials, its fabrication is compatible with the existing complementary metal–oxide semiconductor platform and may find applications in ultracompact and chip‐scale miniature devices, such as optical tweezers operating at nanoscale dimensions.^[^ ^48^, ^63^, ^64^ ^]^

## 2 Results and Discussion

### 2.1 Principle of Broadband Achromatic Modulation of the SOC with Cascaded Metasurfaces

**Figure 1:**
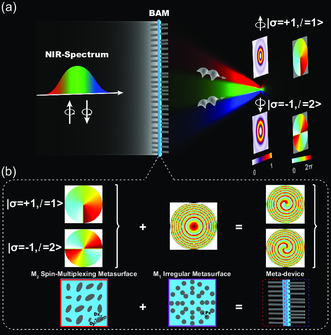
Schematic illustration of broadband achromatic modulation of the SOC with cascaded metasurfaces. a) Schematic of the BAM operating in transmission mode. Near‐infrared beams with different spin states are normally incident on the cascaded metasurfaces. The transmitted beam can be converted OV beam with different AMs and achromatically focused at the prescribed focal plane. b) The broadband spin‐multiplexing metasurface for performing the SOC can be designed utilizing elliptical birefringent nanopillars with different dimensions ($D_{x}$ and $D_{y}$) and orientation angles (*ϕ*). Broadband achromatic polarization‐insensitive metalens is composed of nanopillars with identical dimensions but variable lattice constant ($P_{x}$), which we refer to as an irregular metasurface.

**Figure** **1**a schematically illustrates the broadband achromatic focusing for the SOC of light via cascaded metasurfaces. It's capable of converting different CP beams into the focused OV beams with distinct and spin‐controlled topological charge number on the predesigned focal plane. To introduce the focused chromatic aberration correction effect into broadband OV beams carrying spin‐controlled AMs (See Note S1, Supporting Information), the desired phase spectrums for the SOC should be functions of spatial coordinate (x,y) and angular frequency *ω*.

(1) $$\varphi^{|\sigma ,l\rangle }(r,\theta ,\omega )=-\frac{\omega }{c}(\sqrt{{\left(r\right)}^{2}+{\left(F_{0}\right)}^{2}}-\sqrt{{\left(r_{0}\right)}^{2}+{\left(F_{0}\right)}^{2}})+l^{\sigma }\theta ,\;(\sigma =\pm 1)$$

where $F_{0}$, $r_{0}$, and $l^{\sigma }$ are focal length, reference position, and topological charge number for different spin intrinsic state, respectively. $r=\sqrt{x^{2}+y^{2}}$ and $\theta =\text{arctan}\frac{y}{x}$ are radial and azimuth coordinates. ω and c are angular frequency and the speed of the light, respectively. |σ,l⟩ represents the CP OV beams with different AMs. A single hybrid phase‐based metasurface can be capable of realizing monochromatic spin‐multiplexing focusing, but generally fails to modulate broadband dispersion due to low polarization conversion efficiency.^[^ ^43^, ^59^ ^]^ The chromatic dispersion mainly arises from the resonant phase dispersion of the building meta‐atoms and the intrinsic dispersion of the materials that are used to construct the meta‐atoms. To achieve the desired broadband focused OV phase spectrums in Equation (1) and focused broadband incident beams achromatically, the phase‐dispersion term used for achromatically focusing should be decoupled from the dispersionless spin‐multiplexing spiral profiles for the SOC in Equation (1). By the means of method of separation of variables, Equation (1) can be rewritten as

(2) $$\varphi^{|\sigma ,l\rangle }(r,\theta ,\omega )=\varphi (r,\omega )+\varphi^{|\sigma ,l\rangle }(\theta )$$

Herein, a high‐efficiency broadband achromatic polarization‐insensitive metalens is proposed through elaborately engineering the phase‐dispersion spectrum φ(r,ω) in Equation (2) based on irregular metasurface (denoted as M1 shown in Figure 1b). The dispersionless spin‐multiplexing spiral profiles, $\varphi^{|\sigma =1,\text{l}=1\rangle }(\theta )$ and $\varphi^{|\sigma =-1,\text{l}=2\rangle }(\theta )$ can be realized via a single spin‐multiplexing metasurface (denoted as M2) with high‐broadband polarization‐conversion efficiency. By cascading M1 and M2 into a compact BAM with the desired focused spiral profiles, as shown in Figure 1b, high polarization‐conversion and focusing efficiency ensure the successful realization of broadband achromatic‐focused OV beams with switchable AMs driven by the SOC of light. More details are discussed in Sections 2.2 and 2.3, respectively.

### 2.2 High‐Efficiency Broadband Achromatism with Irregular Metasurfaces

**Figure 2:**
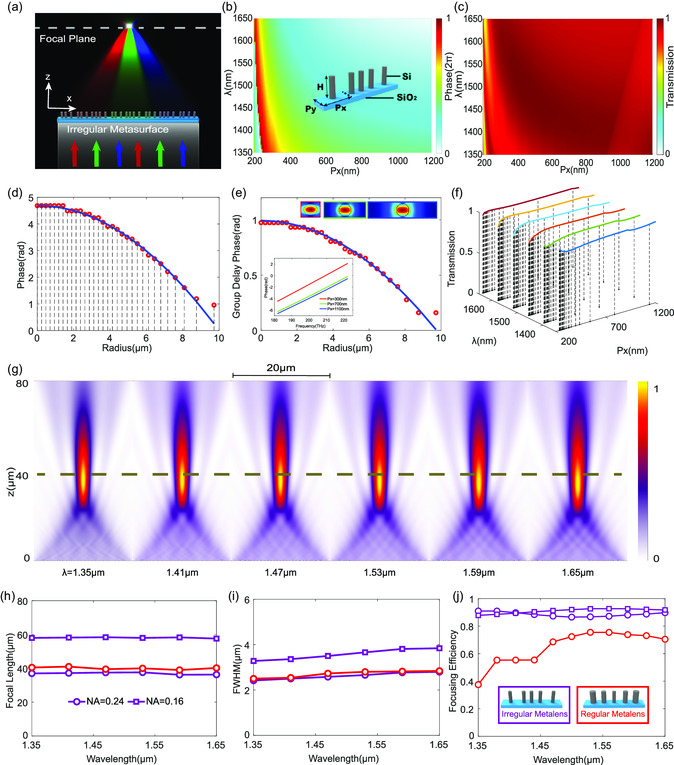
High‐efficiency broadband achromatic modulation with irregular metasurfaces. a) Schematic of the irregular broadband achromatic metasurface operating in a transmission mode. The designed irregular metasurface can efficiently engineer the dispersion and achromatically focus a broadband near‐infrared beam on predesigned focus. b, c) Phase and transmittance databases of irregular meta‐atoms. The meta‐atom is made of a circular amorphous silicon nanopillar (with fixed radius *r* = 200 nm and height *H* = 1400 nm) deposited on a silica rectangular lattice (with varying *P*_*x*_ and fixed *P*_*y*_ = 250 nm) shown in the inset in c. d) The realized and required phase distributions for focusing. e) The realized and required group delay phase spectrum for controlling the dispersion. The inset shows the guiding‐mode profiles and the corresponding phase spectra of the selected meta‐atoms. f) The transmission spectrum of the selected meta‐atoms for the design of the irregular achromatic metasurface. g) Simulated intensity distributions along *x*–*z* cross sections for each sampled wavelength. h) The extracted focal length functioning versus wavelength at different NAs. i) The FWHM of the focal spots vary with the wavelengths sampled at different NA. j) The focusing efficiency of the irregular metalens for comparison with that of the regular metalens.

As shown in **Figure** **2**a, the broadband achromatic irregular cylindrical metalens is composed of circular silicon nanopillars with the fixed radius of r=200 nm but variable lattice constants $P_{x}$ (shown in the inset of Figure 2b). Compared with regular metasurfaces (generally constructed by nanopillar phase shifters with varying dimensions and identical lattice constant), the designed irregular metasurface is capable of avoiding resonance effects to guarantee high‐broadband transmission efficiency and linear phase dispersion. First, we perform a parameter sweep of the nanopillars by varying lattice constants $P_{x}$ for *x*‐polarized incidence in a continuous‐wavelength range from 1.35 to 1.65 μm using finite‐difference time‐domain method (simulated details shown in Experimental Section). Figure 2b,c shows the extracted transmittance and the phase spectrum as a function of $P_{x}$ ranging from 200 to 1200 nm, from which the phase dispersion can be obtained for broadband achromatic focusing shown in Figure 2a. For comparison, the phase and transmission spectra for the regular meta‐atoms (with the same height of *H* = 1400 nm and optimal lattice constant of 615 nm) are provided in Figure S1, Supporting Information. The near‐unity broadband transmission and full 2*π* phase spectrum confirm the high broadband performance of the proposed irregular metasurface, when compared with the regular ones.

By combining Equation ((1), (2)), one can obtain the mapping between the phase dispersion imposed by the subwavelength meta‐atom with specified lattice constant $P_{x}$ and the modulated phase dispersion φ(r,ω) at each pixel coordinate in the irregular metasurface. Subsequently, the cylindrical broadband achromatic metalens with the diameter of *D* = 20 μm and numerical aperture (NA) of 0.24 is constructed for *x*‐polarization incidence. As shown in Figure 2d,e, optimal meta‐atoms with specified phase compensation can be chosen to construct the broadband achromatic metasurface by the means of optimized filtering strategy (See Note S2, Supporting Information). The strong guiding mode confinement in the nanopillar and the linear phase dispersion (shown in the inset of Figure 2e) indicate that the wavefront manipulation with the irregular meta‐atoms is a localized and nonresonate effect. Shown in Figure 2f, near‐unity broadband transmission of the selected meta‐atoms further reveals the validity of high‐efficiency achromatic methodology via the irregular metasurface configuration.

Figure 2g shows the simulated intensity distributions for the metalens with NA = 0.24 along the axial plane, in which the dashed line marks the position of the focal plane. The simulations confirm the broadband achromatic focusing behavior of the irregular metalens. Besides, we have also designed an achromatic metalens with NA = 0.16 for confirming the validity of our achromatic approach (more simulated results are provided in Figure S2, Supporting Information). Through analyzing the intensity distributions of *x*‐*z* cross sections for each sampled wavelength, Figure 2h summarizes the focal lengths and indicates small derivations from −2.5% to 2.5% relative to the mean focal length at each sampled wavelength. The results further confirm that the irregular metalens successfully implements achromatic focusing over a continuous wavelength from 1.35 to 1.65 μm. In addition, we also characterized the quality of the focal spots for each sampled wavelength (the *x*‐cuts intensities across the focal spots can be seen in Figure S2, Supporting Information). The extracted full width at half maxima (FWHMs) for all the sampled wavelengths are summarized in Figure 2i. The results exhibit the realization of the nearly diffraction‐limited focal spots. The focusing efficiency is shown in Figure 2j. The focusing efficiencies of irregular metalens over 85% across the entire designed bandwidth identify the realization of high‐efficiency achromatic focusing, compared with that of the regular metalens constructed by the meta‐atoms from the library shown in Figure S1, Supporting Information. Here, the focusing efficiency of the metalens is defined as the ratio of the optical power passing through a circular aperture (with radius 2–3 times the FWHM spanning the center of the focal spot) to the incident power.

**Figure 3:**
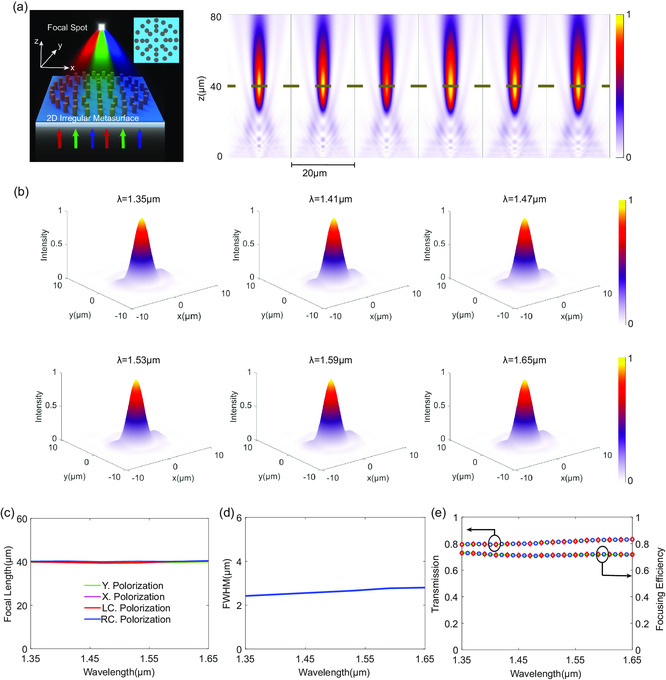
Polarization‐insensitive broadband achromatic focusing with irregular metasurface. a) Schematic of the irregular broadband achromatic metalens and the simulated intensity distributions along *x*–*z* cross sections at each sampled wavelength. The inset shows the top view of the 2D‐circular metalens. The black dashed lines indicate the position of the predesigned focal plane. b) The intensity distribution profiles of the focal spots for each sampled wavelength. c) Focal lengths for different polarization incidence at each sampled wavelength. d) The FWHMs of the focal spots. e) Broadband transmission and achromatic focusing efficiency for different polarizations.

Following the proposed design method for phase‐dispersion engineering, we designed a circular and broadband achromatic polarization‐insensitive metalens (*D* = 20 μm, NA = 0.24) with rotational symmetry for achromatically focusing OV beams with different spin–orbit‐conversion states. **Figure** **3**a shows the schematic of the polarization‐insensitive broadband achromatic metalens and the simulated intensity distribution profiles along the *x*‐*z* cross sections at each sampled wavelength. The corresponding intensity distributions of the *x*‐*y* cross sections along the focal plane (the black dashed lines shown in Figure 3a) at each sampled wavelength are shown in Figure 3b. The results indicate the realization of achromatic and nearly diffraction‐limited focusing with highly symmetric focal spots at each sampled wavelength. The simulated focal lengths for different polarizations are plotted in Figure 3c, which shows a small derivation (maximum variation of 2% relative to the mean focal length) across the entire designed bandwidth. Figure 3d shows the extracted FWHMs for all the sampled wavelengths. As shown in Figure 3e, the focusing efficiencies over 75% across the entire designed bandwidth further reveal the high‐efficiency achromatic focusing, albeit with the reductions due to relatively strong side lobes compared to cylindrical metalens. The nearly identical focal lengths and working efficiencies for different polarizations robustly indicate realization of high‐efficiency polarization‐insensitive broadband achromatic focusing via the proposed irregular metasurface configuration (shown in the inset in Figure 1b).

### 2.3 High‐Efficiency BAM for SAM‐to‐OAM Conversion with Switchable AM

To achieve the SOC of light, the key issue is the design of metaatoms capable of independently controlling the two output spin eigenstates (σ=±1) and accurately introducing the helical‐phase profiles $\varphi^{|\sigma ,\text{l}\rangle }(\theta )$ defined by Equation (2). In this work, the required spin‐multiplexing metasurface consisting of birefringent meta‐atoms is in the employment of engineering the SOC. In order to independently impart phase profiles on the two output spin eigenstates respectively, the Jones matrix J(x, y) that describes light–metasurface interaction can be expressed as

(3) $$\left\{ \begin{array}{c} J(x,y)|\sigma =+1,\;l=1\rangle =e^{i\varphi |\sigma =+1,\;l=1\rangle (\theta )}|\sigma =-1\rangle \\ J(x,y)|\sigma =‐1,\;l=2\rangle =e^{i\varphi |\sigma =‐1,\;l=2\rangle (\theta )}|\sigma =+1\rangle \end{array}\right.$$

where $|\sigma =\pm 1\rangle =\left[\begin{array}{c} 1\\ \pm i \end{array}\right]$ represent the Jones vectors of the spin eigenstates.

**Figure 4:**
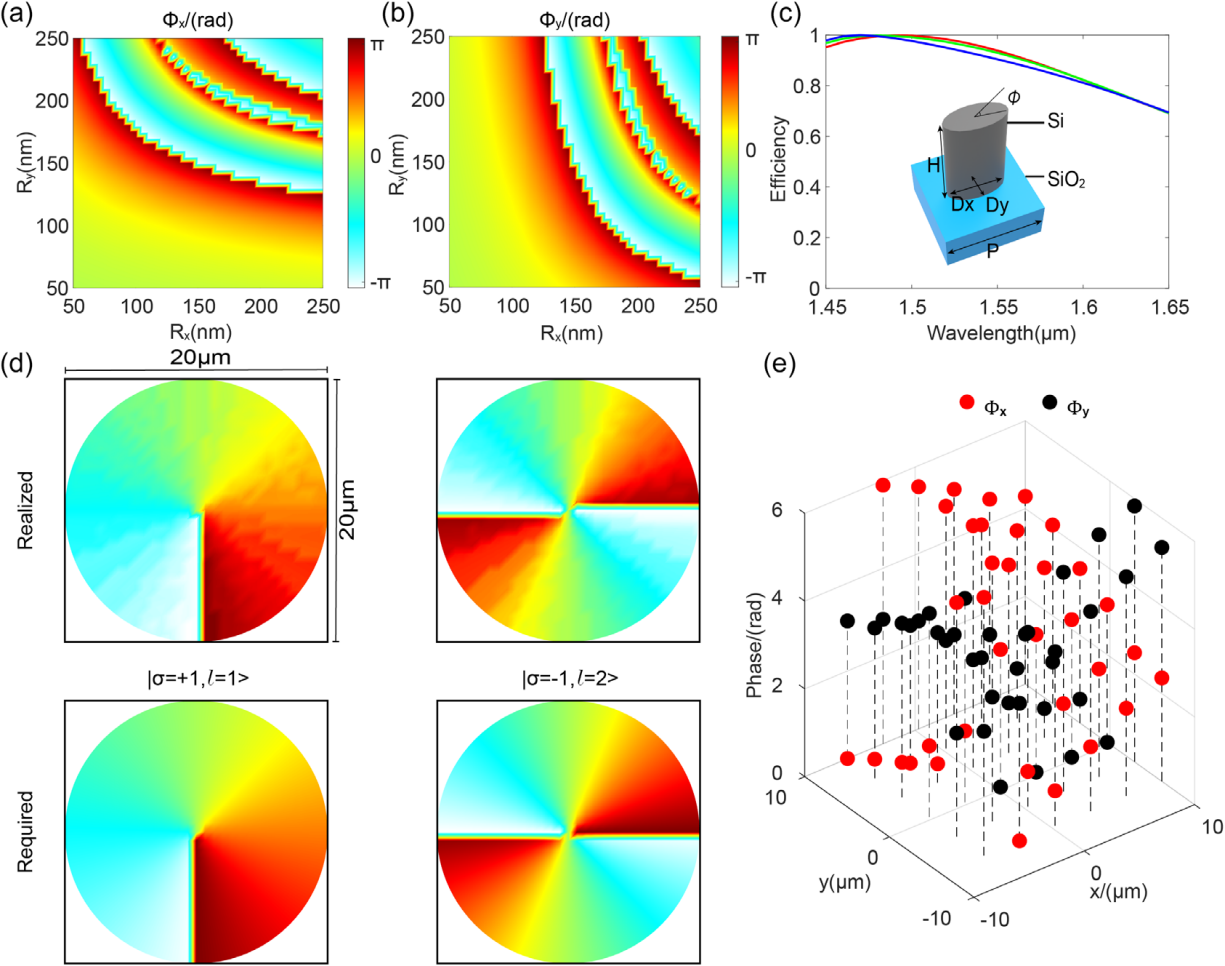
SAM‐to‐OAM with tunable topological charge number. a, b) Phase maps of nanopillars as a function of the major‐axis *R*_*x*_ and minor‐axis *R*_*y*_ for *x*‐ and *y*‐polarizations, respectively. c) Polarization conversion efficiencies across the entire designed bandwidth for the selected meta‐atoms. The inset represents the structural configuration of the meta‐atom. d) The realized and required phase profiles for tunable SOC states with different topological charge numbers. e) Phase shifts for *x*‐ and *y*‐polarized incidences for the selected and optimal meta‐atoms used in the design of the SOC meta‐device with switchable topological charge number.

For the spin‐multiplexing metasurface M2 consisting of silicon elliptical nanopillars shown in the inset of **Figure** **4**b, the optical response of the birefringence meta‐atoms can be described by the Jones matrix as

(4) $$\;J(x,y)=R(-\phi (x,y))[\begin{array}{cc} e^{i{\text{Φ}}_{x}(x,y)} & 0\\ 0 & e^{i\Phi_{y}(x,y)} \end{array}]R(\phi (x,y))$$

where $\Phi_{x}\left(x,y\right)$ and $\Phi_{y}\left(x,y\right)$ denote the spatial propagation phases for the birefringence meta‐atom at (x,y) under *x*‐ and *y*‐polarizations along two symmetry axes. ϕ(x,y) denotes the orientational angle of the meta‐atom, which determines the geometric phase shift. *R* is a 2×2 rotation matrix which can be expressed as$\;R\left(\phi \right)=\left[\begin{array}{cc} \cos\phi & \sin\phi \\ -\sin\phi & \cos\phi \end{array}\right]$. Based on FDTD simulations, we calculate the phase maps of the meta‐atoms across the entire designed bandwidth ranging from 1450 to 1650 nm (simulated details shown in methods sections).

Figure 4a,b shows the simulated phase shifts $\Phi_{x}$ and $\Phi_{y}$ of the elliptical meta‐atoms as a function of the semimajor axis (*R*_*x*_) and semiminor axis (*R*_*y*_) under *x*‐ and *y*‐polarized incidence with wavelength of 1500 nm, respectively. The high polarization conversion efficiencies (average value of 85%) across the entire designed bandwidth are shown in Figure 4c, which ensures the realization of high‐performance broadband SOC metadevice with switchable topological charge number.

By combining Equation ((1), (2), (3), (4)), spin‐decoupled broadband phase control can be achieved (detail derivations shown in Note S3, Supporting Information). The most appropriate set (Rx, Ry,ϕ) of the meta‐atom at the corresponding pixel position can be selected from the meta‐atom library (shown in Figure 4a,b) by minimizing an error function defined as the maximum error between the required phase and the simulated phase profile based on Equation (S10) in Note S3, Supporting Information. Based on the strategy of broadband spin‐decoupled dispesionless phase control, we have designed the broadband spin‐multiplexing metasurface M2 (as shown in Figure 1b). Figure 4d shows the realized phase profiles conducted by M2 operating at the two SOC states, |σ=+1,l=1⟩ and |σ=−1,l=2⟩. They are in agreement with the theoretically required ones (shown the bottom in Figure 4d). The results indicate that the spin‐multiplexing metasurface can perform the SOC operation on circular‐polarized incidence. To satisfy the half‐wave plates condition from Equation (S10), the meta‐atoms with phase difference of close to *π* between the *x*‐ and *y*‐polarization can be selected, shown in Figure 4e.

**Figure 5:**
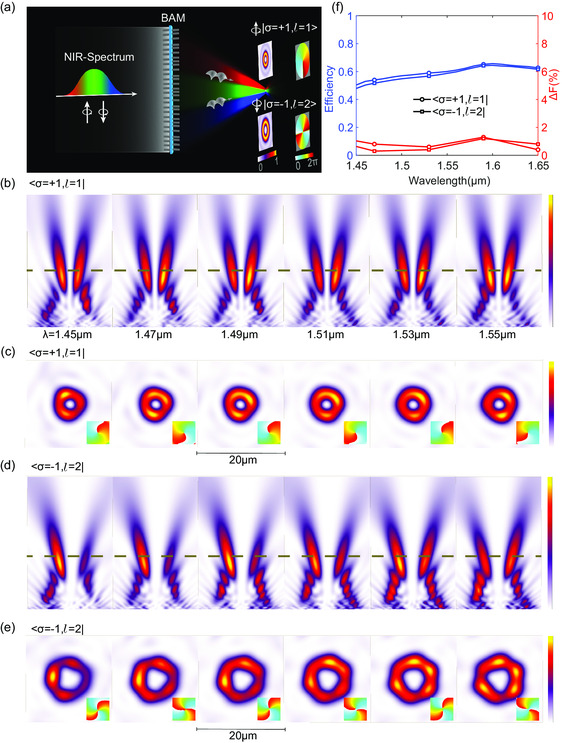
BAM for engineering the SOC with tunable topological charge number driven by the SOC. a) Schematic principle of the broadband achromatic SOC metadevice. The broadband achromatic SOC metadevice can convert different broadband CP beams into the achromatic focused OV beams with distinct and spin‐controlled topological charge number on the predesigned focal plane. b,d) Simulated intensity distribution profiles along *x*–*z* cross sections for different SOC states |σ=+1,l=1⟩ and |σ=−1,l=2⟩, respectively. c,e) The corresponding focal plane intensity profiles for the different SOC states. The insets show the spin‐controlled spiral phase distributions with tunable topological charge number for the achromatic focused OV. f) The broadband efficiency and focal length shifts for the designed bandwidth.

Focused OV beams with tunable properties in multiple dimensions are highly desirable in modern photonics, particularly for wide operating bandwidth and tunable topological charges. As schematically depicted in **Figure** **5**a, the ultracompact BAM with the diameter of *D* = 20 μm has been successfully accomplished through elaborately cascading the proposed M1 and M2 (the thickness of the intermediate spacer between the two layers is 2 μm). The realized spiral phase distributions with the M2 and the focused OV phase profiles are demonstrated in Figure S4, Supporting Information. The high achromatic focusing performance and efficiency ensure the efficient decoupling between the focused phase dispersion and SOC defined by Equation (1). As a result, the BAM is capable of implementing the generation of broadband achromatic focused OV beam with switchable AM driven by the SOC of light. It can convert the broadband near‐infrared beams into the OV beam with photonic spin‐controlled topological charge number, and the photons are achromatically concentrated at the predesigned focus.

We perform 3D‐finite‐difference time‐domain (FDTD) simulations for characterizing the performance of the designed BAM. Figure 5b,d shows the simulated intensity distributions of *x*‐*z* cross sections at each sampled wavelength for different SOC states, |σ=+1,l=1⟩  and |σ=−1,l=2⟩, respectively. It can be seen that hollow‐shaped OV beams with centers on almost the same focal lengths are generated, respectively. The simulated focal lengths are almost constant (maximum variation 1.5% relative to the mean focal length) across the designed bandwidth ranging from 1450 to 1650 nm, as shown in Figure 5f. We have also simulated the focal lengths sampled at different thicknesses of the intermediate spacer for comparison in Figure S4, Supporting Information. The results confirm the design of the BAM in our work. To better illustrate the focused OV beams driven by the SOC of light, Figure 5c,e shows the corresponding focal plane intensity profiles along the dotted lines shown in Figure 5b,d. The doughnut‐shaped focal spots with significant SOC‐dependent sizes reveal that the proposed BAM successfully implements the achromatically focusing and tunable SOC functionality (more simulations can be seen in Figure S3, Supporting Information). Besides, the focused helical phase profiles in the central region of the focus further validate the achromatic focused OV beam with switchable AM on‐demand driven by the SOC of light (shown in the insets of Figure 5c,e). As shown in Figure 5f, the broadband focusing efficiency up to 60% robustly confirms the realization of broadband high‐efficiency BAM, which promises potential applications in integrated optical trapping at nanoscale dimensions.^[^ ^63^, ^64^ ^]^ Here, the efficiency of the BAM is defined as the ratio of the optical power of the focused CP beam to that of the incidence with opposite helicity.

## 3 Conclusion

In summary, we demonstrated a general high‐efficiency broadband achromatic methodology to implement spin‐controlled multifunctional achromatic metadevice for switchable spin‐orbit‐conversion states based on the cascaded metasurface platform. Through efficiently decoupling the SOC from the modulation of phase dispersion, the metadevice is capable of simultaneously implementing achromatic focusing and SOC‐multiplexing functionality. With this platform, we successfully accomplish the generation of broadband achromatic focused OV beam with switchable SOC state and on‐demand topological charge number. The broadband efficiency up to 60% further validates our achromatic approach for control of the dispersion and SOC of light. We believe that the achromatic focused and tunable structured light driven by photonic SOC proposed here will pave the promising way for AM‐based classical and quantum optical applications. It may provide interesting research directions for deeply understanding light–matter interaction and light control at nanoscale dimensions, benefiting from the ultracompact nature, high focusing efficiency, and easy integration.

## 4 Experimental Section

All numerical simulations were conducted using 3D FDTD simulations. To obtain the database of the building blocks used in metadevice design, periodic boundary conditions were applied along the *x*‐ and *y*‐ axes and perfectly matched layers (PML) was applied along the *z*‐axis (direction of light propagation). For obtaining the phase maps $\Phi_{x}$ and $\Phi_{y}$ in Section 2.3, periodic arrays were illuminated with *x*‐ and *y*‐polarized plane waves within the desired bandwidth, respectively. Also, we adopted the PML boundary condition for all boundaries to carry out the 3D FDTD simulations for the BAMs.

## Conflict of Interest

The authors declare no conflict of interest.

## Author Contributions

K.O. and X.C. conceived the original ideas presented in this work. K.O., L.X., and W.H. developed the theoretical aspect and numerical design. L.X., W.H., and K.O. carried out the numerical simulations and designed the samples. W.H., L.X., and K.O. analyzed the data and discussed the results. The manuscript was jointly written by K.O., W.H., and L.X. K.O., X.C., and Z.W. organized the project, analyzed the results, and prepare the manuscripts. All authors discussed the results and commented on the manuscript.

## Supporting information

Supplementary Material

## Data Availability

The data that support the findings of this study are available from the corresponding author upon reasonable request.
