# Supplementary material for: High‐Efficiency Broadband Achromatic Metadevice for Spin‐to‐Orbital Angular Momentum Conversion of Light in the Near‐Infrared
Source: Small Sci. 2024 Feb 13;4(5):2300273. doi: 10.1002/smsc.202300273 (PMC11935231; doi:10.1002/smsc.202300273)
Supplement: Supplementary file 1 — Supplementary Material [file SMSC-4-2300273-s001.pdf]

Supplementary Information for:

High-efficiency Broadband Achromatic Meta-devices for Spin-to-Orbital Angular Momentum Conversion of Light in the Near-Infrared

Lingyun Xie[1,2,3,4+], Hengyi Wan[1,2,3+], Kai Ou[1,2,3*],Junming Long[1], Zining Wang[1], Yuchao Wang[1], Hui Yang[5], Zeyong Wei[1,2,3*], Zhanshan Wang[1,2,3,6] and Xinbin Cheng[1,2,3,6]

[1] Institute of Precision Optical Engineering, School of Physics Science and Engineering, Tongji University, Shanghai, 200092, China

[2] MOE Key Laboratory of Advanced Micro-Structured Materials, Shanghai, 200092, China

[3] Shanghai Frontiers Science Center of Digital Optics, Shanghai 200092, China

[4] College of Electronic and Information Engineering, Tongji University, Shanghai, 200092, China

[5] School of Physics and Electronics, Hunan Normal University, Changsha, 410081, China

[6] Shanghai Institute of Intelligent Science and Technology, Tongji University, Shanghai 200092, China

[*] Corresponding authors: kaiou211122@tongji.edu.cn, weizeyong@tongji.edu.cn

[+]These authors contributed equally: Lingyun Xie, Hengyi Wan

E-mail: 21310075@tongji.edu.cn (L.Xie), 2130945@tongji.edu.cn (H.Wan)

Supplementary Note 1. Method for the design principle of broadband achromatically focusing

In this work, the phase spectra imposed by the metalenses can be generally expressed as:

$$\varphi^{M1}(x, y; \omega) = \omega t\big(x, y, \chi(\omega)\big) + C(\omega) \tag{1}$$

Here, the parameter $\chi(\omega)$ describes the functionality realized by the metalenses at each modulated polarization state. For instance, $\chi(\omega)$ can be the focal length $F(\omega)$ for the metalens. $C(\omega)$ is an arbitrary spectrum function and determines the reference phase at each frequency point. The group delay is:

$$t_g = \frac{d\varphi^{M1}}{d\omega} = \omega t\big(x, y, \chi(\omega)\big) + \frac{d\mathrm{C}(\omega)}{d\omega} + \omega \frac{dt}{d\chi}\frac{d\chi(\omega)}{d\omega} \tag{2}$$

To achromatically manipulate a broadband incidence, the parameter $\chi(\omega)$ should be dispersionless ($\frac{d\chi(\omega)}{d\omega} = 0, \chi(\omega) = \chi_0$). Thus, the group delay is:

$$t_g(x, y; \omega) = t(x, y, \chi_0) + \frac{d\mathrm{C}(\omega)}{d\omega} \tag{3}$$

Furthermore, the group delay dispersion is $\frac{dt_g}{d\omega} = \frac{d\mathrm{C}(\omega)}{d\omega}$. Obviously, $\mathrm{C}(\omega)$ plays a key role in manipulating the dispersion behavior of the metalens. The choice of $\mathrm{C}(\omega)$ depends on the dispersion of the metaatom. Because of the nearly linear group delay of the metaatom, $\mathrm{C}(\omega) = t_0\omega$ is a proper choice. Thus, we can rewrite Supplementary Equation (4) as:

$$\varphi^{M1}(x, y; \omega) = \varphi^{M1}(x, y; \omega_{min}) + (t(x, y, \chi_0) + t_0)(\omega - \omega_{min}) \tag{4}$$

Finally, at each frequency point, by combining Supplementary Equations (2)-(7), we can obtain the mapping between the polarization-dependent phase-dispersion ($\varphi_n^{M1}(\omega_{min})$, $\frac{d\varphi_n^{M1}(\omega)}{d\omega}$) imposed by the metaatom and the modulated phase dispersion at each pixel. With this mapping function, the metalenses for simultaneously manipulating the phase and phase dispersion behavior of the light wave can be realized. Moreover, the phase profiles for focusing can be expressed as:

$$\varphi^{M1}(x, y; \omega) = \varphi_{lens}(x, y, F_0; \omega) \tag{5}$$

Subsequently, based on the general Snell's law, a general form for $\mathrm{C}(\omega)$ is:

$$\mathrm{C}(\omega) = \frac{\omega}{c}\left(\sqrt{(r_0)^2 + (F_0)^2}\right) \tag{6}$$

Finally, the modulated phase profiles can be recast as:

$$\varphi^{M1}(x, y; \omega) = -\frac{\omega}{c}\left(\sqrt{(r)^2 + (F_0)^2} - \sqrt{(r_0)^2 + (F_0)^2}\right) \tag{7}$$

where F_0, r_0, and r are constant focal length, reference position, the radial and azimuth coordinates, respectively.

Supplementary Note 2. Method for choosing the optimal irregular metaatoms

Using the principle proposed in the main text and **Supplementary Note 1**, we can devise an objective function (the error between the required phasor spectrum and that achieved by the irregular metaatom):

$$\text{Error}(x,y,r_0;n)=\sum_{k=1}^{N}\left|A_t e^{i\varphi^{M1}(x,y;\,r_0;\omega_k)}-A_{meta}(n;\omega_k)e^{i\varphi_{meta}(n;\omega_k)}\right| \tag{8}$$

To select the optimal metaatom at each pixel coordinate (x, y) on the metasurface. We design an optimal algorithm that sweeps over possible r_0 and finds out the best possible set of meta-units for the designed metalenses. The optimal metaatoms can be chosen by minimizing the summation of phasor errors across all design wavelengths and pixel coordinates (x,y). A_t are the amplitudes at 他 the modulated phase spectrum and A_{meta} is achieved by the metaatoms. In this paper, $A_t=0.98$ is an optimized choice. It's worth noting that we sample many closely spaced wavelengths (or frequency points) for approximating the continuous spectrum.

Supplementary Note 3. Method for the design of spin-multiplexing metasurface with birefringence meta-atoms

By combining Equations (3-4) in the main text, for independent control the spiral phase shift profiles of left-handed circularly polarized ($\sigma=+1$) and right-handed circularly polarized components ($\sigma=-1$), The Jones matrix $J(x,y)$ realized by the spin-multiplexing metasurface can be expressed as:

$$J(x,y)=\begin{bmatrix}\dfrac{e^{i\varphi^{|\sigma=+1,l=1\rangle}(x,y)}+e^{i\varphi^{|\sigma=-1,l=2\rangle}}}{2} & \dfrac{ie^{i\varphi^{|\sigma=+1,l=1\rangle}(x,y)}+ie^{i\varphi^{|\sigma=-1,l=2\rangle}}}{2}\\ \dfrac{ie^{i\varphi^{|\sigma=+1,l=1\rangle}(x,y)}+ie^{i\varphi^{|\sigma=-1,l=2\rangle}}}{2} & \dfrac{-e^{i\varphi^{|\sigma=+1,l=1\rangle}(x,y)}-e^{i\varphi^{|\sigma=-1,l=2\rangle}}}{2}\end{bmatrix} \tag{9}$$

Finally, the spin-decoupled broadband phase control with M2 can be derived as:

$$\begin{cases}\varphi^{|\sigma=+1,l=1\rangle}(x,y)=\Phi_x(x,y)-2\phi(x,y)\\ \varphi^{|\sigma=-1,l=2\rangle}(x,y)=\Phi_y(x,y)+2\phi(x,y)-\pi\\ \phi(x,y)=(\varphi^{|\sigma=+1,l=1\rangle}(x,y)-\varphi^{|\sigma=-1,l=2\rangle}(x,y))/4\end{cases} \tag{10}$$

The phase and transmission spectrum and the focusing performance for the regular metasurface for comparison shown in the main text.

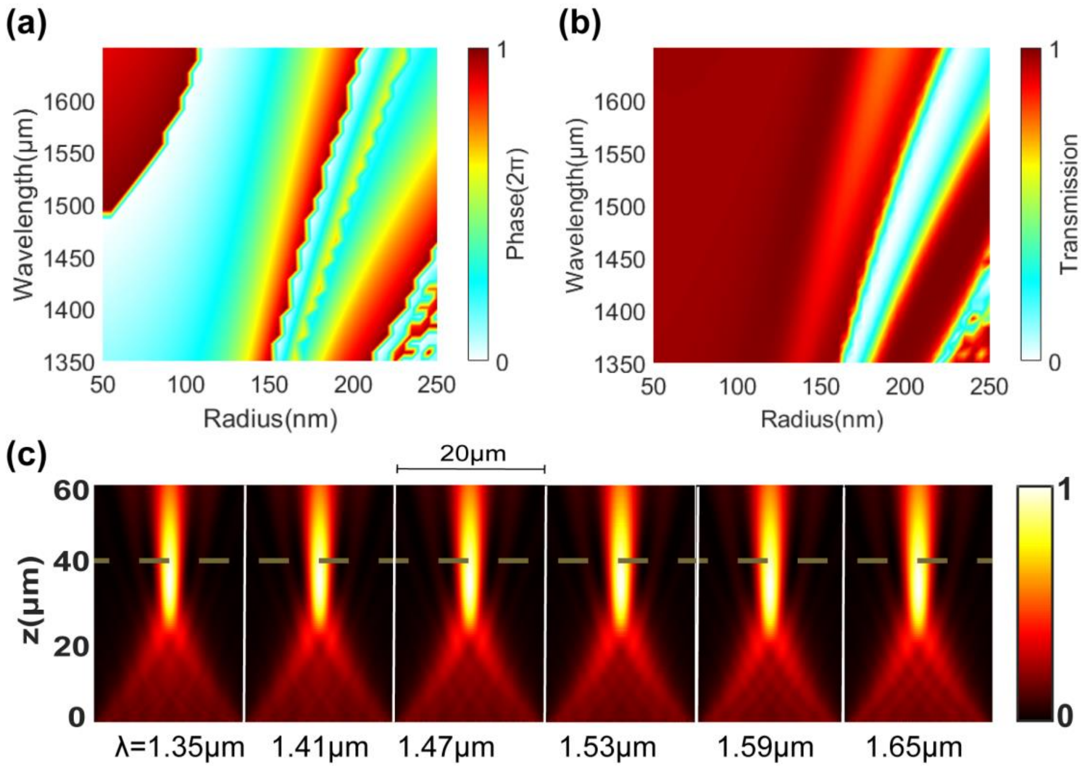

Supplementary Figure S1: The phase (a) and transmission spectrum (b) for the regular metasurface. (c) Simulated normalized axial intensity distributions over the designed wavelength for the regular metalens.

Additional simulation results for high-efficiency achromatic metalens with irregular metasurfaces

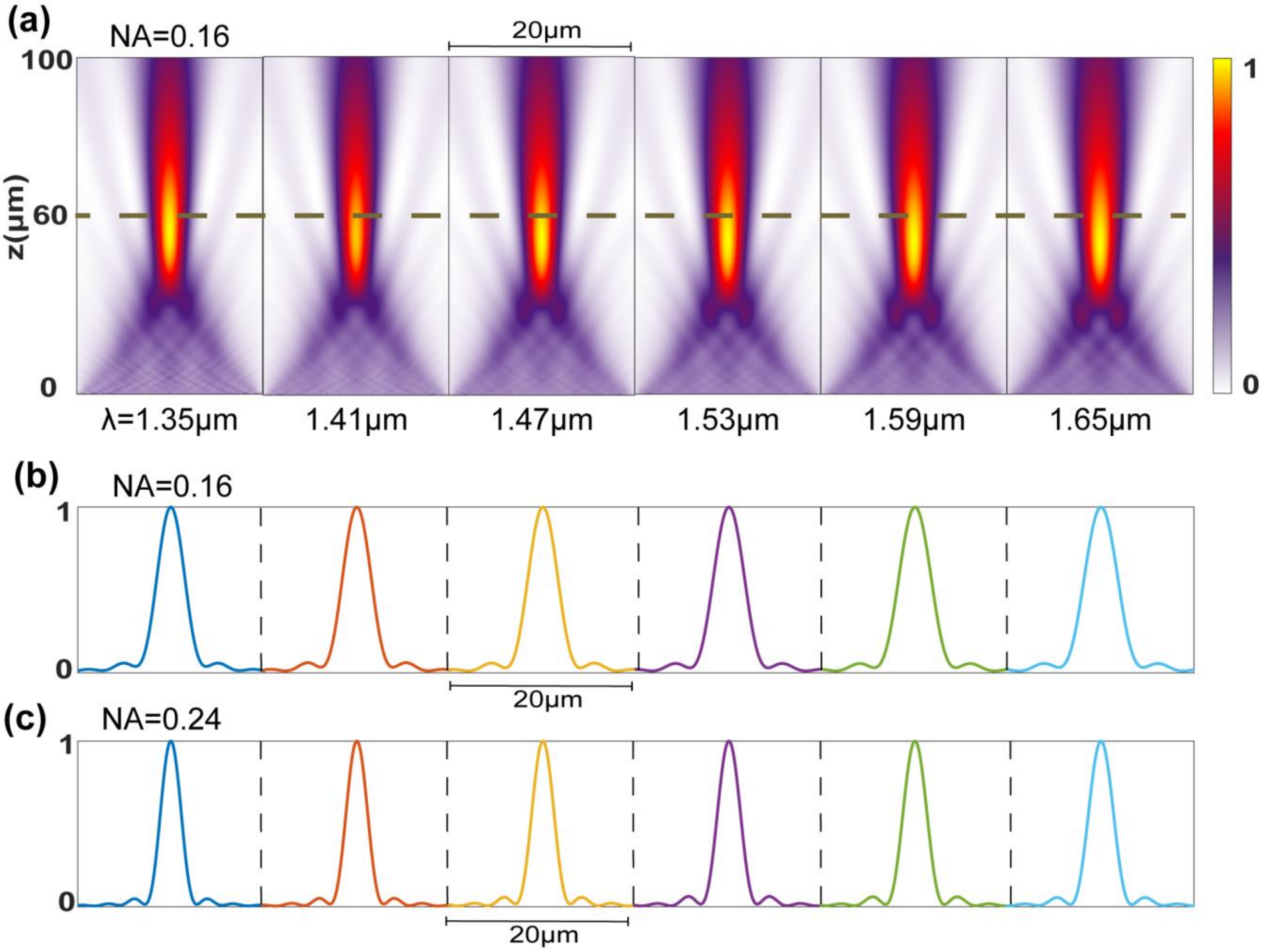

Supplementary Figure S2: Simulated normalized axial intensity distributions and the corresponding x-cut intensity profiles of the focal spots over the designed wavelength for achromatic metalenses.

Additional simulation results of the BAM for modulation of the SOC.

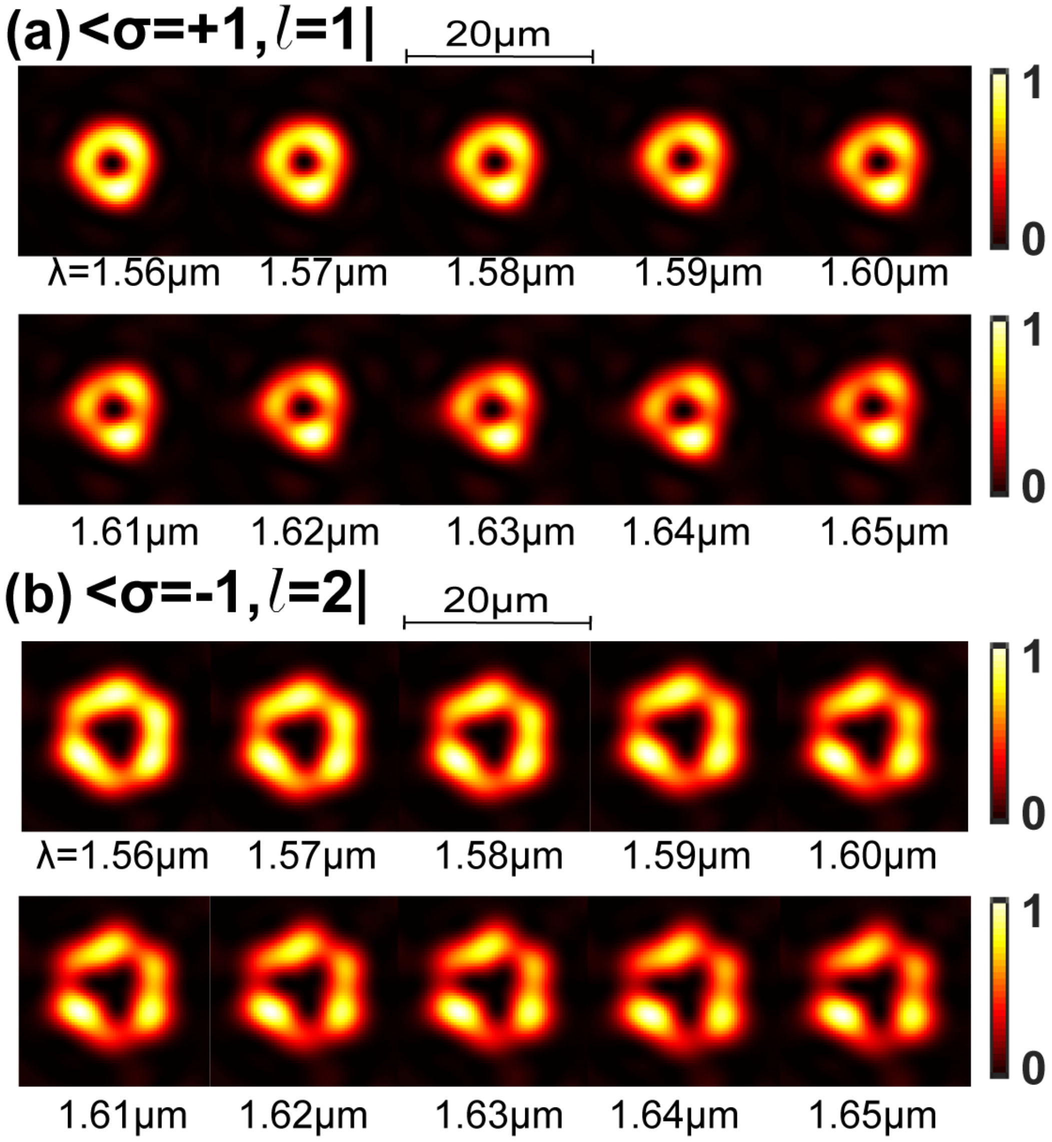

Supplementary Figure S3: Simulated intensity distributions of the focused OV spots over the designed wavelength region ranging from 1.56 to 1.65μm for the BAM.

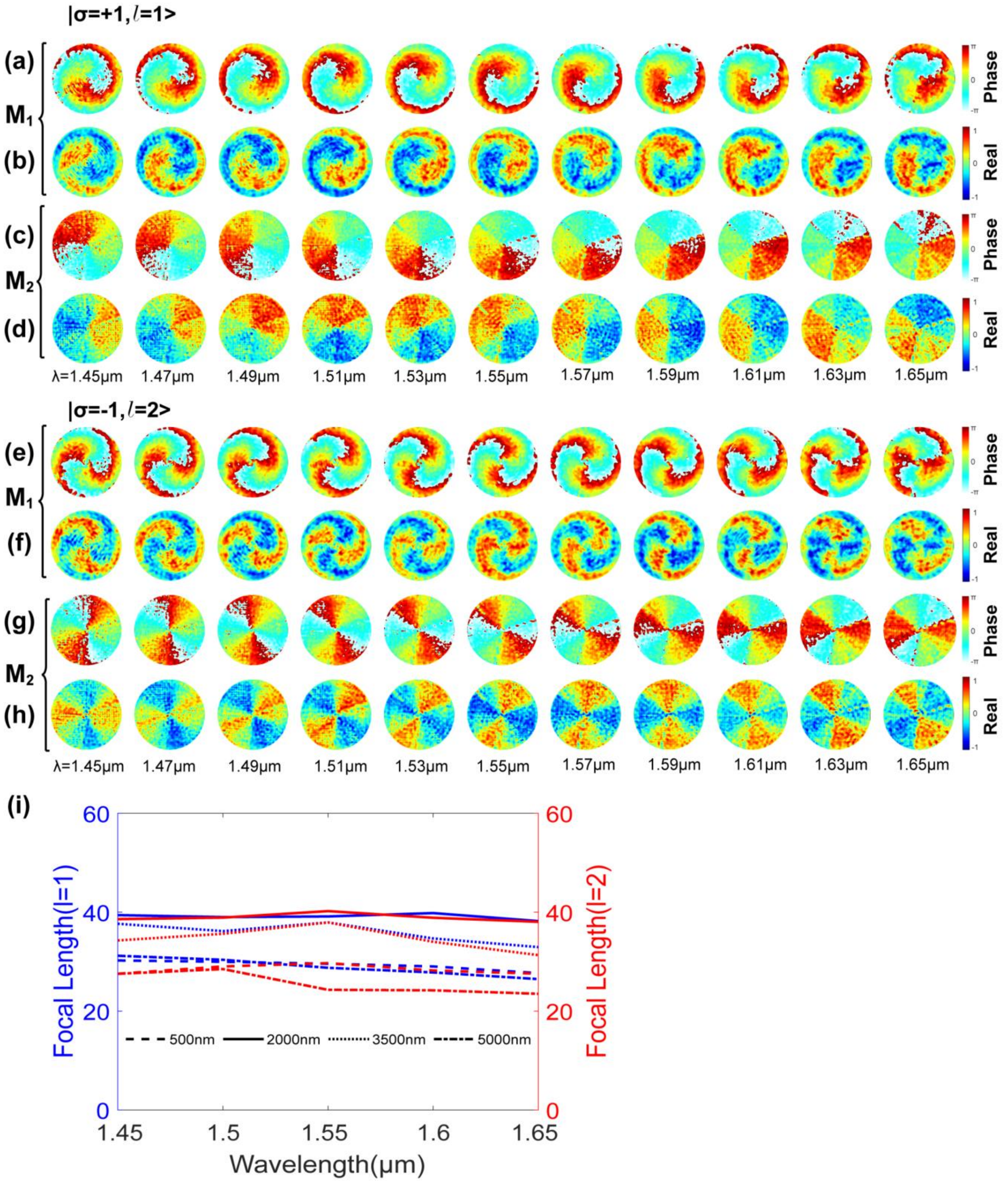

Supplementary Figure S4： (a-d) Simulated the incidence distribution upon the M1 and M2 for $|\sigma=+1, l=1\rangle$. (e-h) Simulated the incidence distribution upon the M1 and M2 for $|\sigma=-1, l=2\rangle$. (i) The focal lengths vary with the wavelengths sampled at different spacer thickness.
